# Implications of Early Diagnosis and Intervention in the Management of Neurodevelopmental Delay (NDD) in Children: A Systematic Review and Meta-Analysis

**DOI:** 10.7759/cureus.38745

**Published:** 2023-05-08

**Authors:** Sarah S Aldharman, Khalid H Al-jabr, Yazeed S Alharbi, Nadyah K Alnajar, Jomanah J Alkhanani, Abdullrahman Alghamdi, Rim A Abdellatif, Abdullah Allouzi, Albaraa M Almallah, Syed F Jamil

**Affiliations:** 1 College of Medicine, King Saud Bin Abdulaziz University for Health Sciences, Riyadh, SAU; 2 College of Medicine, Prince Sattam Bin Abdulaziz University, Al-Kharj, SAU; 3 College of Medicine, Qassim University, Buraydah, SAU; 4 College of Medicine, Almaarefa University, Riyadh, SAU; 5 College of Medicine, Alrayan Medical College, Al-Madinah, SAU; 6 College of Medicine, Sulaiman Al-Rajhi University, Al Bukairiyah, SAU; 7 College of Medicine, Qassim University, Qassim, SAU; 8 Research, King Abdullah International Medical Research Center, Riyadh, SAU; 9 Pediatrics, King Abdullah Specialized Children's Hospital, Riyadh, SAU

**Keywords:** child development, cognitive behavior, diagnosis, intervention, neurodevelopmental delay

## Abstract

Neuro-developmental delay (NDD) is when a child's reflexes and nervous system are underdeveloped or immature at a given stage of child development. Neurodevelopmental delays account for delayed skill development surrounding speech, social, emotional, behavioral, motor, and cognitive delays. NDD might affect the child's psychological and physical well-being, resulting in chronic disease and disabilities throughout adulthood. This review sought to investigate the implication of early diagnosis and intervention of NDD in children. In this regard, this research opted for a systematic meta-analysis that used keywords and Boolean operators to search through main databases, including the Web of Science, JStor, PsychINFO, Science Direct, Cochrane, Scopus, and ASSIA. The result identified that telehealth interventions improved the management of NDD in children. Also, the Early Start Denver Model (ESDM) model was determined to improve the quality of life for NDD children. Another model was LEAP (Learning Experience and Alternative Program for Preschoolers and Their Parents) and Leap (Learning, engaging, and Playing), which improved behavioral, education, and social interventions in NDD children. The study identified that technology could revolutionize NDD interventions in children, possibly improving the quality of life. The parent-children relationship was shown to enhance the management of this condition; thus, it is recommended as one of the best ways to intervene in the management of NDD. Most importantly, the integration of machine learning algorithms and technology can create models; while this may not be significant in the treatment of childhood NDD but instead might be ideal in improving the quality of life for NDD children. Moreover, their social and communication skills along with academic achievements will improve. The study proposes further research in order to understand the different types of NDDs and their intervention strategies to help the researchers identify the most accurate models to improve the conditions and support the parents and guardians in the management.

## Introduction and background

Development delay (DD) refers to a child’s delayed pace of skills development compared to others within the age group [1]. Generally, developmental delays are evident in a child with slowed problem-solving and social skills development [1,2]. Also, children may experience restricted motor skills, language, and speech development [2]. A condition where a child experiences slow development in more than one growth area is called global development delay (GDD) [3]. This systematic review and meta-analysis examined early diagnosis and intervention in managing neurodevelopmental delay (NDD) in children, where neuro-developmental delay (NDD) refers to an underdeveloped or immature reflex and nervous system in a given stage of child development [4]. The immature growth and development of the nervous system interfere with perceptual skills, hand-eye coordination, visual functioning, and motor skills development [5]. Generally, NDD arises from neonatal and perinatal events, such as the maternal factors associated with gestational diabetes [4]. Low birth weight and intrauterine growth restrictions are also leading causes of NDD [3]. Some children get NDD from intrapartum brain injury, including perinatal arterial ischemic stroke, encephalopathy, and hypoxic-ischemic [3]. Neonatal diseases and infections such as listeria, GBS, varicella zoster, and rubella can also potentially cause NDD [6]. Lack of early diagnosis and intervention can escalate the NDD reducing the capacity to optimize the normal functioning of the child [6]. 

NDD potentially impairs the child’s psychological and physical health, and its effects can carry on to adulthood, creating chronic disabilities [7]. The main contributors to NDD include autism spectrum disorders (ASD) and intellectual or global developmental disabilities [7]. Such conditions hijack a child’s normal development causing delays and possible disabilities. World Health Organization suggests that early diagnosis and intervention of the infant at risk of NDD can help mitigate the negative implications and establish productive relationships between the mother and the healthcare providers [8]. Also, early intervention minimizes the escalation of emotional, cognitive, and motor impairments in children [5]. Before the intervention, NDD diagnosis is crucial to formulate the optimal intervention. NDD testing involves brain examination, where brain development is the interplay of the physical and social environment and genes which modulates gene expression and transcription [4,9]. Other NDD tests are neurophysiological tests, including electroencephalography. Finally, NDD testing can be done through neuroimaging using brain magnetic resonance imaging (MRI) and cranial ultrasounds [8,9].

The NDD management process incorporates the role of many individuals in the child’s life [10]. The parents/guardians, social relationships, and environment play critical roles in the early intervention of NDD [4,6]. Some interventions provide parents/guardians with the right education to provide care and interactions to their charges that aim at developing self-efficacy, reducing depressive symptoms, and modifying anxiety to the utmost care. The aim of this study is to explore the implications of the early diagnosis and intervention of NDD in children’s development and parent/guardian interactions.

## Review

Search strategy

This review applied a literature search procedure to identify credible sources about the diagnosis and intervention of NDD in children. The aim was to examine the implications of the diagnosis and intervention of NDD in children’s development and parent/guardian interactions. The review applied inclusion and exclusion criteria to determine the most accurate sources reflecting the research aims and objectives [11]. In this respect, the inclusion criteria considered several characteristics to qualify the study for reference in this review [11]. The first criterion is the language of publication; this review only considered studies written in English. The second criterion was examining the nature of the publication; only journal articles were included. The third criterion was the year of publication; this study considered studies conducted from 2007 to 2023 and studies published before and during 2007 were excluded from this study. This study aimed at using relevant and current sources reflective of the early diagnosis and intervention management strategies in the current NDD development. The search process used keywords and Boolean operators “AND” or “OR” to diversify the scope and identify important sources in proving the aim and the objectives [11]. The following keywords were utilized for this study (diagnosis, neurodevelopmental delays, intervention, early, management, children, early intervention and diagnosis, management of NDD in children).

The keywords were applied in the following databases to help identify accurate and verifiable journal articles to create table characteristics and substantiate the findings [11]. The databases included the Web of Science, JStor, PsychINFO, Science Direct, Cochrane, Scopus, and ASSIA. Another factor considered in this review was the inclusion of studies with full text to quantify the credibility and reliability of the sources in justification of this research aim and objectives.

Data extraction

Data extraction involved transcribing information from the sources to qualify for the review. Reviewing data before extraction aimed to increase accuracy and reduce bias. This research conducted a robust data extraction process involving much paper, including RevMan 5.4 software (Cochrane Collaboration, London, UK). This research investigated the implication of early diagnosis and intervention in managing NDD in children. Therefore, data extraction applied the keywords and the Boolean operators to analyze the participant numbers and characteristics. In this respect, the intervention and the control group participants were recorded for the randomized clinical trials to improve the analysis. In addition, the extraction process captured the research objective, the comparison, and the outcome, which also included the comments on the implications of this study.

Quality assessment

Quality was of the essence in this review, which entailed systematically and carefully examining the research to identify its relevance, value, and trustworthiness [12]. The following questions were ideal in the quality appraisal process. First, whether the study was focused on addressing the query and whether the methodology was valid to address the question, whether the question was biased concerning the study design, and whether the entire approach was addressed. Also, the precision of the findings concerning the confidence interval and the results obtained. Lastly, the quality appraisal considered external validity, which examined whether the results were valid in addressing the study population.

Analysis

The included data were analyzed using RevMan 5.4 software, where risk difference, a test of heterogeneity, forest plot, and funnel plot is discussed in length concerning the table characteristics. 

PRISMA flowchart

**Figure 1:**
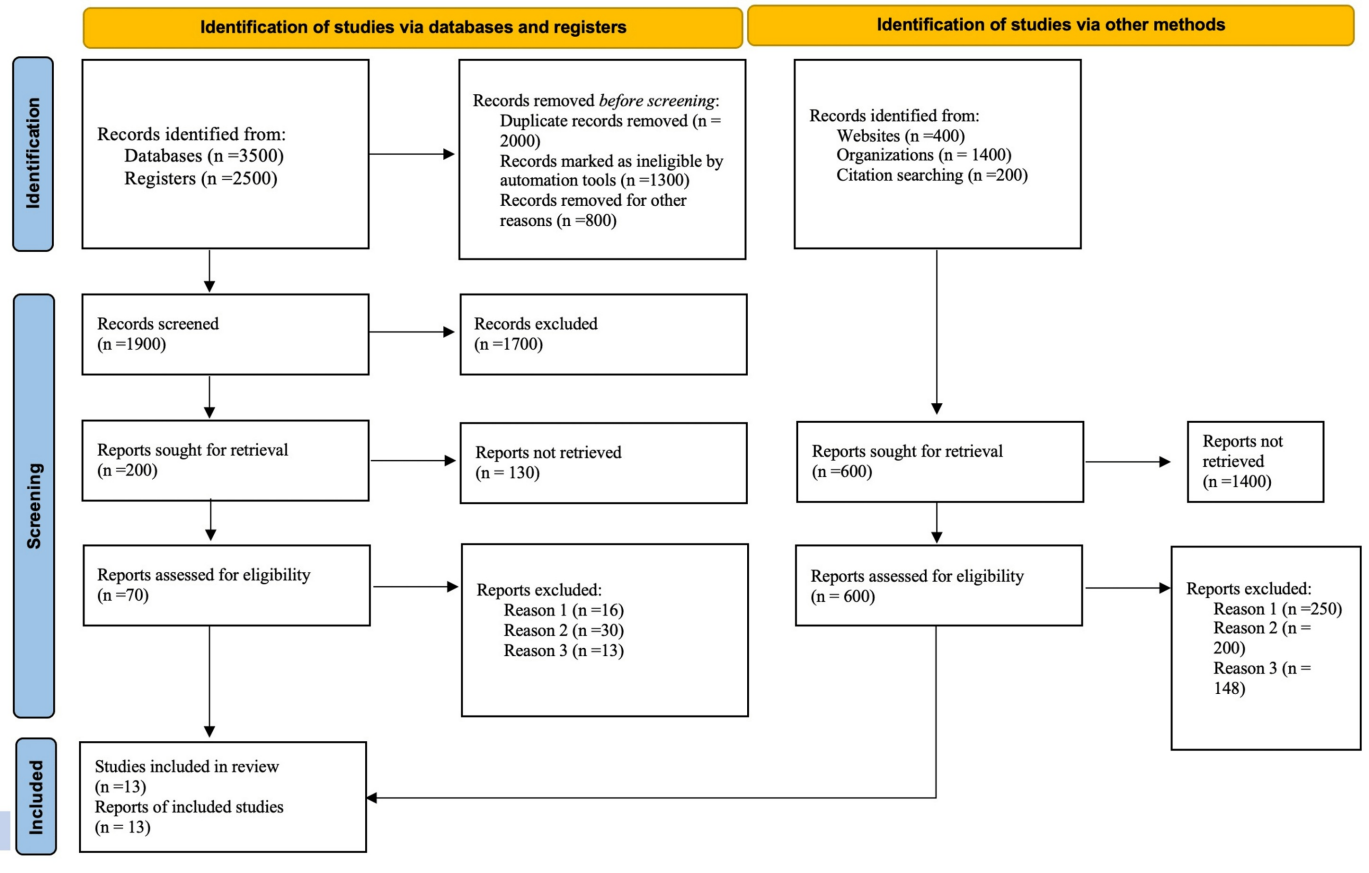
PRISMA Flow Diagram

PRISMA flowchart is used in systematic meta-analysis reviews to support the transparent and easy-to-understand search strategy [13]. The inclusion criteria and keywords were crucial in accumulating important data in establishing the implications of early diagnosis and intervention on children with neurodevelopmental delays [13]. Using the Boolean “AND” and “OR” keywords, the study identified 3500 records from databases and another 2500 from registers. Before the screening procedure, 2000 duplicated records were removed, 1300 were automatically marked as illegible using automation tools, and 800 records were removed following irrelevance from the study. Only 1900 records qualified for screening, where 1700 records were excluded based on English language and year of publication, where the inclusion criteria settled for studies published after 2007. Two hundred papers were sought for retrieval, where 130 sources did not meet the threshold due to a lack of complete text to assess the quality. Only 70 sources were evaluated for eligibility, whereas 16 sources were unavailable, and 30 were excluded because they contained children’s developmental delays that touched on the nervous and body systems. Also, 13 citations were banned due to unclear objectives and outdated references. Only 12 sources were identified through the databases and registers. The search strategy identified papers from other methods as well, including 400 sources identified from websites, 1400 from organizations, and 200 from citation searching. After this, 1400 sources were removed, leaving 600 for consideration. Then 250 studies were removed due to a lack of clear objectives and methodologies, 200 could not be correlated with the research question, and 148 did not have clear information regarding the interventions and the outcome. As a result, only 13 sources for included in this study. In this respect, the visual depiction of the results is shown in Figure 1 below.

Study characteristics

The characteristics in Table 1 provided the studies the author(s) included, year of publication, study design, objectives, and implications in the diagnosis and interventions of NDD in children. Also, the table consists of the nature of the impact that defines the intervention’s strength in managing NDD.

**Table 1:** Characteristics of the included studies NDD: neuro-developmental delay; ESDM: Early Start Denver Model; ASD: autism spectrum disorder; LEAP: Learning Experiences and Alternative Program; LEaP: Learning, Engaging, and Playing; PND: post-natal depression; BN: Bayesian network; FCT: functional communication training

Author(s) &year	Study design	Objective	Implications	Nature of Implication
Cavonius-Rintahaka et al., 2022 [14].	Randomized clinical trials	To compare the ordinary clinical treatment and the dialogical family guidance in the diagnosis and management of NDD	Dialogical family guidance effectively strengthened social support, psychoeducation, practical advice, and managing daily life.	Effective
Dawson G et al., 2009 [15].	Randomized controlled trials	To evaluate the efficacy of the ESDM in the behavioral intervention for (ASD).	The ESDM model showed efficacy in the reduction of ASD severity and the improvement of the adaptive and cognitive behavior in ASD	Efficient
Eapen et al., 2013 [16].		To assess the effectiveness of the ESDM model for preschool children in community settings	Early use of the ESDM model as an intervention for ASD children provided significant economic and clinical benefits.	Significant
Strain and Bovey, 2011 [17].	Clustered randomized design	To investigate early intervention of autism spectrum disorders using the LEAP model.	LEAP model significantly improved autism symptoms and social, language, and cognitive behavior for preschool children.	Significant
Denis et al., 2022 [18].	Observatory study	To detect Malo, a mobile app for the recognition of PND and NDD	Malo was an algorithm-based mobile application that proved efficient in diagnosing PND and NDD in children.	Efficient
Hatakenaka et al., 2022 [19].	Retrospective cohort study	To assess if the BN model can predict early motor development problems among children.	The BN is not a sensitive screening tool but can be used for early assessment of developmental motor delays in NDD.	Not sensitive
Sujatha and Jain, 2016 [20].	Prospective cohort study	To estimate the incidence of NDD in preterm babies in India due to perinatal risks	Preterm babies are at risk of NDD, requiring early intervention programs.	Effective
Armstrong et al., 2021 [21].	Randomized control trial	To investigate LEaP (Learning, engaging, and Playing) to improve the outcome for children with developmental delays.	LEaP improved goal achievement and family support for children with NDD.	Improves
Hwang et al., 2013 [22].	Single-blinded randomized control trial	To assess the effectiveness of a routine-based approach for early intervention for children with NDD.	The study observed that routine-based early intervention was effective in the family-selected goals and promoting functional outcomes.	Effective
Roberts and Kaiser, 2015 [23].	Randomized controlled trials	To test the effects on language outcomes of a caregiver-implemented communication intervention targeting toddlers at risk for persistent language delays	Early language intervention improved language development delays.	Improves
Valentine et al., 2021 [24].	Systematic review	To assess the application of telehealth in neurodevelopmental disorders.	Telehealth effectively monitored NDD, decreasing diagnosis time and increasing treatment availability.	Effective
Kingsdorf and Pančocha, 2021 [25].	Scoping review	To examine the behavioral telehealth practices for families and children with neurodevelopmental delays.	Telehealth was considered viable for behavior-analytic support of children and families impacted by neurodevelopmental delays.	Viable
Kasparian et al., 2022 [26].	Randomized controlled trials	To measure the impact of FCT in telehealth for children with NDD	FCT-enabled telehealth was an efficient behavioral treatment for children with NDD caused by Fragile X syndrome	Efficient

Meta-analysis

The following section provides a meta-analysis to establish the implications of early diagnosis and intervention in managing neurodevelopmental delays in children. The subsequent studies were the most influential in showing the results during the intervention and the outcome. 

Forest plot

**Figure 2:**
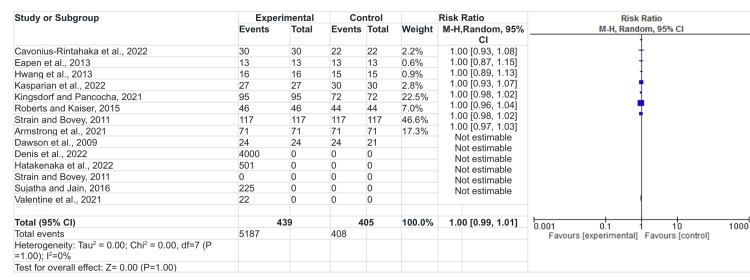
Forest plot

The forest plot as shown in Figure 2 below provides a clear interpretation of the findings of this study [14-26]. The columns indicate the included studies, the intervention group, the control group, the weight, and the outcome effect in the numerical and graphical format [27]. The graphic display in the forest plot represents the study at the box as the estimate of the effect, also known as the mean difference, the odd ratio, or the risk ratio [27]. The box size represents the study’s weight; in this representation, studies by [17] carry the most significant weight. It has the largest sample size in comparison to other randomized controlled trials. The sample size represents the population under study and thus [17] carried more weight in establishing the implications of LEAP (Learning Experience and Alternative Program) for preschool children with autism symptoms and social, language, and cognitive behavior development delays. According to [17], using the LEAP program for preschool significantly managed the social, language, and cognitive behavior of children with NDD [25]. A scoping review carried weight in improving the viability of telephone for behavioral intervention for children diagnosed with NDD. Also, another study by [21] was significant in this research by displaying that the LEaP program, which encompassed learning, engaging, and playing, is influential in managing developmental delays in children. Generally, the forest plot shows the studies crossing the vertical line, which means that the null value is within the 95% confidence interval, and thus, there was no statistical difference observed from the control groups to the treatment groups [27].

Risk difference

The diamond box shows the averaged combination of studies within the 95% confidence interval, which means that the studies fall within the vertical line and, thus, no statistical difference between the intervention and the control group, although Table 1 above provides the nature of the implication where some of the intervention improves the management of the NDD in children [28]. Mainly, the studies displayed some improvement in the management and diagnosis of NDD, which also depended on the level of parent education and social, economic, and environmental status surrounding the children with NDD. The chi-square I2 statistic is equally essential in the meta-analysis. An I2 indication of the direction of the analysis depends on the confidence interval used, the chi-square, and the p-value. In this regard, the chi-square is less than 50%, which shows no vital heterogeneity in the studies. The lack of statistical evidence of heterogeneity is not enough to prove that the above-included studies lack variability [28]. Mainly, studies conducted through systematic analysis carry bias from the original author. Even though there is proof of no heterogeneity, the studies are close and could provide a clear picture of the implications of early diagnosis and intervention in the management of NDD among children. 

**Figure 3:**
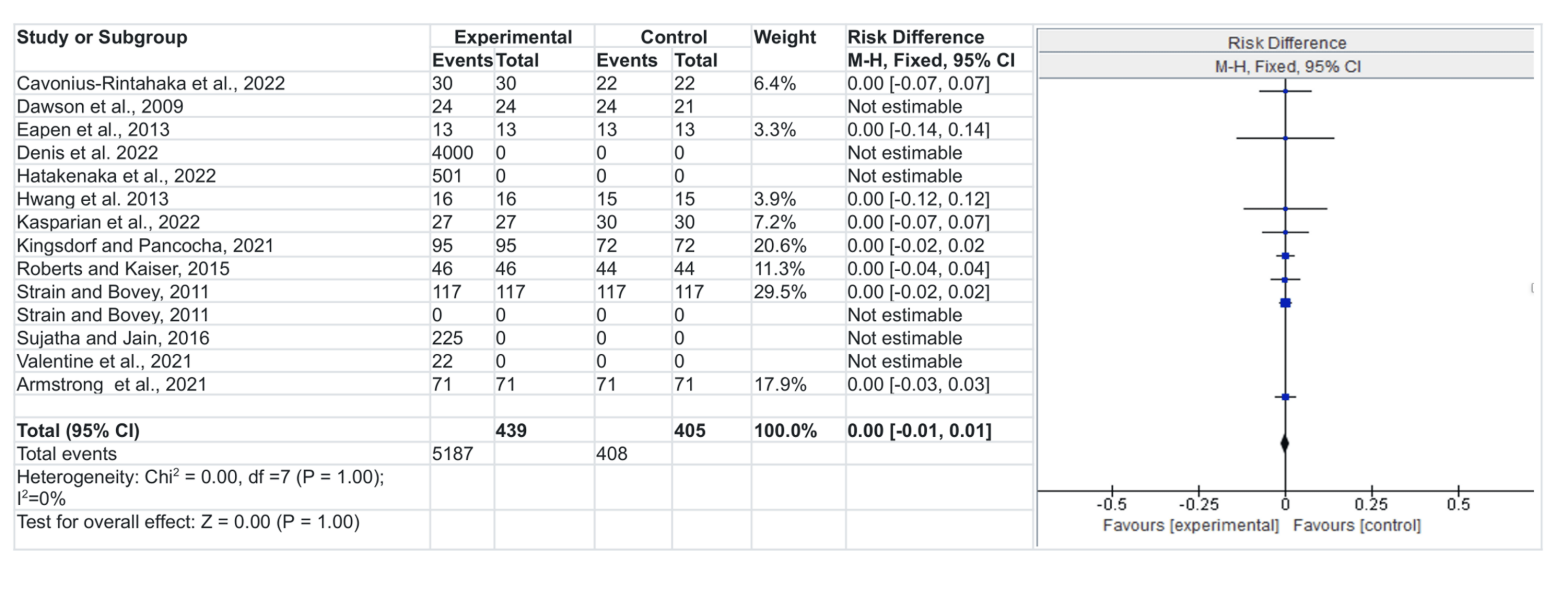
Risk difference

Figure 3 provides a negative risk difference, meaning that early intervention and diagnosis reduced the risk of severe NDDs in children [14-26]. In other words, a negative risk difference means that it was easy to manage NDD children following early intervention and diagnosis. 

Funnel plot

**Figure 4:**
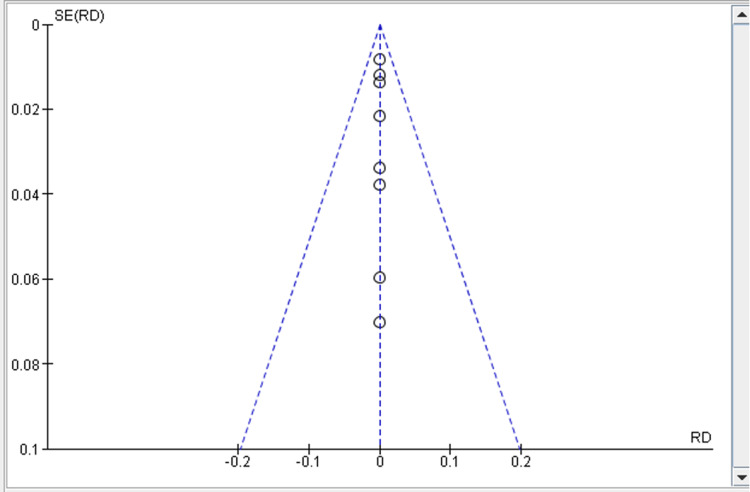
Funnel plot

The funnel in meta-analysis is used in estimating the risk of publication bias within the study [29]. The scattering of the studies within the funnel plot depends on the size of the studies, where the more extensive studies have scattered widely while in small studies the scatter is intact within the treatment effect. All the studies are within the funnel plot, which shows no publication bias existing within the studies, as shown in Figure 4 below [29].

Risk of bias

Another critical measure is the risk of bias in randomized controlled trials [30]. The risk of bias as shown in Table 2 is assessed by considering the judgment of unclear, low, or high in reporting, attrition, performance, and selection [30]. Selection bias arises from the inadequate generation of the study while reporting bias means the selective outcome of the study [30]. The performance bias occurs due to the blinding of the participants’ effects; detection bias is due to measures used, while the attrition bias checks the completeness of the data in projecting the study outcome [30]. Generally, the risk of bias was considerably low; thus, the studies were influential in launching the study’s outcome. 

**Table 2:** Risk of bias

Study authors	Risk sequence generation	Allocation (selection bias)	Blinding of participant bias	Blinding of outcome assessment	Incomplete outcome data (Attrition bias)	Selective reporting (Reporting bis)	Other potential bias
Armstrong et al., 2021 [21].	Low	Low	Low	Unlikely	Low	Low	Unlikely
Cavoniuas-Rintahaka et al., 2022 [14]	Low	Low	Unknown	Medium	Medium	Low	Unlikely
Dawson et al., 2009 [15]	Low	Low	Low	Low	Low	Low	Unlikely
Eapen et al., 2013 [16]	Medium	Low	Low	Unlikely	Moderate	Low	Unlikely
Hatakenaka et al., 2022 [19]	Low	Low	Low	Low	Low	Low	Unlikely
Hwang et al. 2013 [22].	Medium	Low	Low	Low	Low	Low	Unlikely
Kasparian et al., 2022 [26]	Low	Low	Low	Low	Low	Low	Unlikely
Kingsdorf and Pancocha, 2021 [25]	Low	Low	Low	Low	Low	Low	Unlikely
Roberts and Kaiser, 2015 [23]	Moderate	Low	Low	Low	Low	Low	Unlikely
Sujatha and Jain, 2016 [20]	Low	Moderate	Low	Low	Low	Low	Unlikely
Valentine et al., 2021 [24]	Low	Low	Unlikely	Unlikely	Unlikely	Low	Unlikely

Discussion

This study aimed to investigate the implications of early diagnosis and treatment on managing NDD in children. A study was conducted to compare standard clinical treatment with dialogical family guidance in diagnosing and managing NDD [14]. The effectiveness of dialogical family guidance was observed since it strengthened social support and opened a dialogue for practical guidance and managing children with NDD. Also, a similar study incorporated the LEaP model for learning, engaging, and playing to improve NDD outcomes [21]. It was observed that strengthening family relationships and encouraging communication promoted functional outcomes in NDD management [14,21]. In 2013, a similar model applied a routine-based approach whose intervention significantly promoted family-selected goals and functional outcomes in NDD children [22]. As a result, it was proposed to use a family-related dialogical model that strengthens social relationships and communication within the family in establishing functional outcomes for NDD [14,21,22].

Technological advancement has revolutionized different sectors of the globe, and healthcare is one of the most affected by the technologies. The rise of pandemics and instabilities surrounding the health sector has strengthened the use and adoption of technology to address prevalent and emerging conditions. As a result, this study witnessed telehealth’s help in improving and managing NDD conditions. For instance, a recent review showed effective improvement in ND monitoring through telehealth [24]. Moreover, it showcased the viability of telehealth in behavioral management for children with NDD [25]. Also, a study displayed the efficiency of telehealth in functional communication and training of children with NDD, however, it only accounted for NDD caused by Fragile X syndrome [26]. Also, this research identified extensive recommendations for using the Early Start Denver Model (ESDM) for effectively managing behavioral delays in children with autism spectrum disorders. For instance, the effectiveness of the ESDM model for treating and managing developmental delays arising from autism spectrum disorder was noted [15,16]. A study identified an algorithm-based mobile application named Malo to effectively diagnose and recognize NDD and post-natal depression (PND) [18]. Also, a model-based study proposed using the Bayesian network (BN) model to predict early motor development problems for delayed children [19]. An essential factor identified in the quest to manage NDD in children is the use of mathematical models and machine learning algorithms to create models and applications that help the children navigate the physical and emotional environment depending on the nature of NDD. For instance, World Health Organization (WHO) provided a report detailing the importance of assistive technology in covering services and systems for delivering assistive services and products [31]. Using technology to assist children with developmental delays could help improve functioning, well-being, and independence [31]. For instance, memory aids, pill organizers, prostheses, spectacles, communication aids, wheelchairs, and hearing aids are all adequate [31]. Therefore, the use of technology can provide greater results in the management of developmental delays for learners. The technology should contain learning programs and algorithms that can help the children keep up growth in the different segments affecting their life. Our results showed that parent-mediated interventions effectively improved the outcomes of children with ASD in various domains. This finding is consistent with previous research that has shown the effectiveness of parent-mediated interventions for children with ASD [32]. Parent-mediated early intervention was found to have positive impacts on children's communication skills and social-emotional development [33]. Moreover, telephone coaching intervention was effective in improving the viability of parent-child interactions. A systematic review found that telehealth interventions can improve outcomes for children with ASD [34].

Neurodevelopmental delays are among the most challenging conditions affecting many children globally. The study has shown that delays can be managed using assistive models and technology and that none of the applications can effectively reverse the conditions. Therefore, future research on neurodevelopmental delays can help inform the field of medicine on interventions parents can make to prevent future neurodevelopment conditions. The use of screening tools in the population requires careful complementary and multidisciplinary clinical evaluation to enable early detection and intervention to optimize normal functioning.

## Conclusions

The current systematic review offers a narrative summary of the available evidence in this area. Studies have shown that early diagnosis and intervention of neurodevelopmental delays in children can have significant implications for their long-term outcomes. Early diagnosis and intervention through methods such as developmental screening and early intervention services can help children receive the support and therapies needed to improve their outcomes. The study has shown that the delays can be improved using assistive models and technology, but none of the applications can effectively fully reverse the conditions.
